# Supplementary material for: Asymmetry and redundancy of STAT5 paralogs across CD8+ T cell differentiation states
Source: Commun Biol. 2026 Apr 4;9:529. doi: 10.1038/s42003-026-09999-9 (PMC13083952; doi:10.1038/s42003-026-09999-9)
Supplement: Supplementary file 1 — Supplementary Information [file 42003_2026_9999_MOESM1_ESM.pdf]

Title: Asymmetry and redundancy of STAT5 paralogs across $CD8^+$ T cell differentiation states

Authors: Svetlana Ristin [1,2] *, Molly Dalzell [1,2] *, Christopher Armstrong [1], Nisa Ilsin [1], Antonio M. Fontanella [1], Luis Nivelo [1,2], Lothar Hennighausen [3], John J. O'Shea [4] † and Alejandro V. Villarino [1,2] † @

Affiliations:

1 Department of Microbiology and Immunology, Miller School of Medicine, University of Miami, Miami, FL, USA
2 Sylvester Comprehensive Cancer Center, University of Miami, Miami, USA
3 National Institute of Diabetes, Digestive and Kidney Diseases, National Institutes of Health, Bethesda, MD, USA
4 Lymphocyte Cell Biology Section, Molecular Immunology and Inflammation Branch, National Institute of Arthritis, Musculoskeletal and Skin Diseases, National Institutes of Health, Bethesda, MD, USA

* These authors contributed equally

† These authors jointly supervised this work

@ Corresponding author: Alejandro V. Villarino (alejandro.villarino@miami.edu)

Abstract:
Fostering STAT5 signaling is key to immunotherapies that leverage $CD8^+$ T cell biology. Using mouse models, we demonstrate that the two mammalian STAT5 paralogs, STAT5A and STAT5B, are at once redundant and functionally distinct in $CD8^+$ T cells. Thus, they are *asymmetric paralogs*, exhibiting both widespread homology at molecular level and functional asymmetry at cellular level, with STAT5B emerging as dominant. For mechanisms, we determined that STAT5B is twice as abundant, accounting for two-thirds of the total STAT5 pool, and present evidence that it also has distinct, paralog-specific properties. We also defined cytokine- and cell state-restricted STAT5B functions and devised a core signature that spotlights key downstream properties and serves as bioinformatic probe. Together, these studies affirm the centrality of STAT5 in $CD8^+$ T cells, reveal common and circumscribed activities for STAT5A and STAT5B, and present a unifying model that foregrounds both redundancy and asymmetry.

Brief: STAT5 paralog dominance and redundancy in $CD8^+$ T cells

Supplementary Information
7 supplementary figures, 7 supplementary data:

Supplementary Figure 1. STAT5 deficiency markedly impacts $CD8^+$ T cells.
Supplementary Figure 2. Outgrowth of effector and memory $CD8^+$ T cells in STAT5-deficient mice.
Supplementary Figure 3. Asymmetric expression of STAT5 paralogs in $CD8^+$ T cells.
Supplementary Figure 4. Asymmetric function of STAT5 paralogs in $CD8^+$ T cells.
Supplementary Figure 5. Genes associated with only STAT5A or STAT5B are not paralog specific.
Supplementary Figure 6. Core STAT5 signature assembly and validation.
Supplementary Figure 7. Core STAT5 signature applied to scRNA-seq data.
Supplementary Data 1. Gene set catalogue
Supplementary Data 2. Gene set usage
Supplementary Data 3. Low amplitude DEG
Supplementary Data 4. ChIP-seq peak catalogue
Supplementary Data 5. *In vitro* culture conditions
Supplementary Data 6. Replicate and Statistics catalogue
Supplementary Data 7. Plot Supplementary Data

Supplementary Figure Legends

Fig. S1. *STAT5 deficiency markedly impacts CD8⁺ T cells.* (A) Flow cytometry contour plots show surface CD3ε and CD19 proteins on live cells from pLN of *Stat5a/b* $^{flox/flox}$ *Cd4*-Cre$^{+/-}$ mice and littermate controls. (B) Box plots compile frequencies of CD3ε$^+$ T cells, CD3ε$^+$ CD4$^+$ T cells and CD3ε$^+$ CD8$^+$ T cells in mLN, pLN and spleens. Replicate counts and statistical tests for all experiments are listed in Supplementary Data 6.

Fig. S2. *Outgrowth of effector and memory CD8$^+$ T cells in STAT5-deficient mice.* (A-B) Box plots compile frequencies of naïve, memory and effector CD8$^+$ T cells in (A) spleens or (B) bone marrow of STAT5 'allele' mice. (C) Box plots compile frequencies of naïve and effector CD8$^+$ T cells in pLN and spleens of *Stat5a/b* $^{flox/flox}$ *Cd4*-Cre$^{+/-}$ mice and littermate controls. Replicate counts and statistical tests for all experiments are listed in Supplementary Data 6.

Fig. S3. *Asymmetric expression of STAT5 paralogs in CD8$^+$ T cells.* (A) Donut plots relay percentage of total mRNA accounted for by STAT5A or STAT5B in mouse CD8$^+$ Tnv (top row) or Tem/cm cells (bottom row) per ImmGen. (B) Mouse CD8$^+$ Tcm cells from pLN were pulsed with cytokines for 1h. Donut plots relay percentage of p-STAT5 or STAT5 mRNA accounted by STAT5A or STAT5B in total CD8$^+$ T cells (left), CD8$^+$ Tnv (center) or CD8$^+$ Tcm (right) downstream of IL-7 (top row) or IL-15 (bottom row). (C) Flow cytometry histograms show total STAT5 protein levels in total CD4$^+$ and CD8$^+$ cells of the indicated genotypes (Ctl = Isotype control). (D) Donut plots relay percentage of total mRNA accounted for by STAT5A or STAT5B in human CD4$^+$ (top) or CD8$^+$ (bottom) Tnv cells per DICE. (E) Flow cytometry histograms show total STAT5 protein levels across the lymphoid compartment in spleens of WT mice. Replicate counts and statistical tests for all experiments are listed in Supplementary Data 6.

Fig. S4. *Disparities between STAT5A and STAT5B deficient CD8$^+$ T cells.* (A) Flow cytometry contour plots show surface CD44 and CD62L on CD8$^+$ T cells from pLN. Naïve cells are defined as CD44^{low} CD62L^{high} (upper left), central memory cells as CD44^{high} CD62L^{high} (upper right) and effector cells as CD44^{high} CD62L^{low} (lower right). These were the markers and definitions used to sort CD4$^+$ and CD8$^+$ cells for RNA-seq (Fig. 4 and Fig. 7). (B) Positively regulated DEG were subjected to hypergeometric testing against the indicated databases (per Fig. 4C). Scatter plot shows enrichment q values for cytokine regulated genes across genotypes, cytokines and cell states (Y axis; blue = AAB versus WT, orange = BBA versus WT). All gene sets are catalogued in Supplementary Data 1 and usage detailed in Supplementary Data 2. Ex vivo culture conditions detailed in Supplementary Data 7. Replicate counts and statistical tests for all experiments are listed in Supplementary Data 6.

Fig. S5. *Genes bound only by STAT5A or STAT5B are not paralog specific.* (A-C) Venn plots compare DEG mobilized by CA-STAT5A in STAT5A-deficient CD8$^+$ T cells (DEG from Fig. 6A) to Peak-Associated Genes (PAG) linked to (A) STAT5A alone, (B) STAT5B alone or (C) both STAT5A and STAT5B (PAG from Fig. 5D). Genome browser histograms show representative examples. All STAT5A and STAT5B bound regions are catalogued in Supplementary Data 4. All gene sets are catalogued in Supplementary Data 1 and usage detailed in Supplementary Data 2. *Ex vivo* culture conditions for retroviral transduction are detailed in Supplementary Data 5. Replicate counts and statistical tests for all experiments are listed in Supplementary Data 6.

Fig. S6. *Core STAT5 signature assembly and validation.* (A) Stacked bar plot enumerates DEG in CD8$^+$ Tnv or Tcm cells relative to WT controls (blue stack = AAB versus WT; orange stack = BBA versus WT; DEG same as Fig. 4). (B-C) Venn plots compare DEG mobilized by (B) IL-7 or (C) IL-15 in naïve and memory CD8$^+$ T cells. (D) Stacked bar plot enumerates DEG in CD4$^+$ or CD8$^+$ T cells relative to WT controls (plot same as Fig. 7D).

(E-F) Venn plots compare DEG mobilized by IL-7 in (E) naïve or (F) memory T cells. (G) Positively regulated DEG from (D) were subjected to hypergeometric testing against the indicated databases (per Fig. 4C). Scatter plot shows enrichment *q* values for top STAT5-regulated pathways (X axis) across genotypes, cytokines, lineages and cell states (Y axis; blue = AAB versus WT, orange = BBA versus WT). (H) Heat map shows log2 fold change values for all elements of the core STAT5 signature in $CD8^+$ T cells across genotypes, cytokines and cell states. All gene sets catalogued in Supplementary Data 1 and usage detailed in Supplementary Data 2. Ex vivo culture conditions are detailed in Supplementary Data 5. Replicate counts and statistical tests for all experiments are listed in Supplementary Data 6.

Fig. S7. *Core STAT5 signature applied to scRNA-seq data.* (A) scRNA-seq UMAP projection segregates $CD8^+$ T cells responding to acute or chronic LCMV infection. Clustering and annotation are as published. Feature plots show module score enrichment for constituents of the 85 gene core STAT5 signature. (B) Feature plots show module score enrichment for unabridged (top) and top 50 (bottom) positive DEG from the indicated cytokine/state pairs. (C) Module score enrichment for unabridged (top) and top 50 (bottom) negative DEG. (D) Violin plots show enrichment of positive DEG sets within individual cells, across clusters. All gene sets catalogued in Supplementary Data 1.

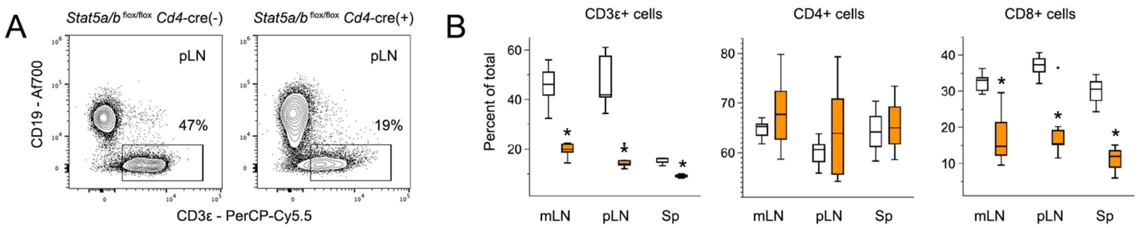

A
Stat5a/b flox/flox Cd4-cre(-)
Stat5a/b flox/flox Cd4-cre(+)
pLN
47%
19%
CD19 - Af700
CD3ε - PerCP-Cy5.5
B
CD3ε+ cells
CD4+ cells
CD8+ cells
Percent of total
mLN
pLN
Sp

Figure S1

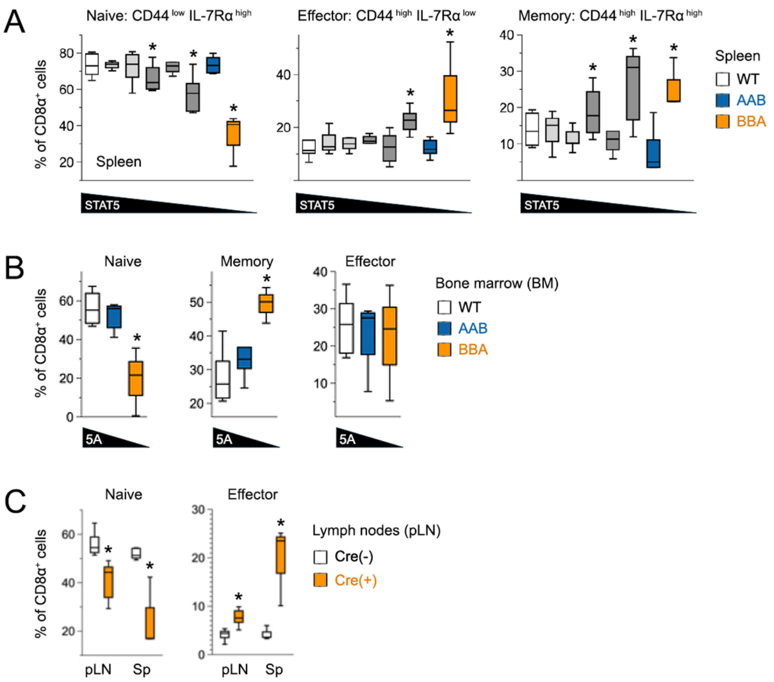
A
Naive: CD44 low IL-7Rα high
Effector: CD44 high IL-7Rα low
Memory: CD44 high IL-7Rα high
% of CD8α+ cells
Spleen
STAT5
Spleen
WT
AAB
BBA
B
Naive
Memory
Effector
5A
Bone marrow (BM)
WT
AAB
BBA
C
Naive
Effector
pLN
Sp
Lymph nodes (pLN)
Cre(-)
Cre(+)

Figure S2

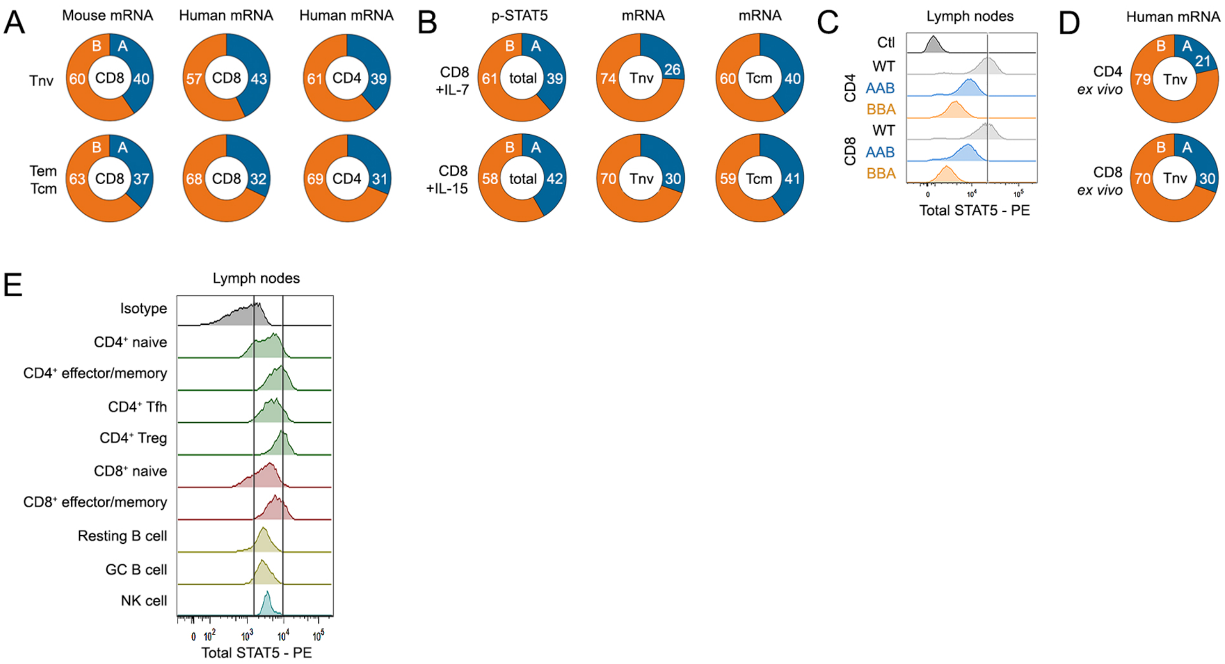
A
Mouse mRNA
Human mRNA
Human mRNA
Tnv
Tem
Tcm
B
A
CD8
CD4
60
40
57
43
61
39
63
37
68
32
69
31
B
p-STAT5
mRNA
mRNA
CD8
+IL-7
CD8
+IL-15
total
Tnv
Tcm
61
39
74
26
60
40
58
42
70
30
59
41
C
Lymph nodes
Ctl
WT
AAB
BBA
CD4
CD8
Total STAT5 - PE
D
Human mRNA
CD4
ex vivo
CD8
ex vivo
79
21
70
30
E
Lymph nodes
Isotype
CD4⁺ naive
CD4⁺ effector/memory
CD4⁺ Tfh
CD4⁺ Treg
CD8⁺ naive
CD8⁺ effector/memory
Resting B cell
GC B cell
NK cell
Total STAT5 - PE

Figure S3

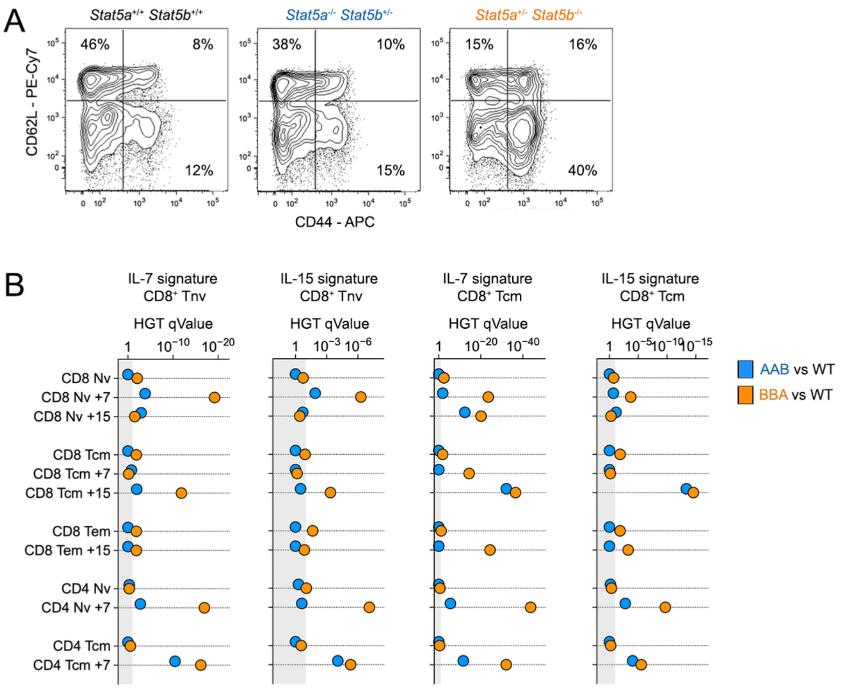

A
Stat5a+/+ Stat5b+/+
Stat5a-/- Stat5b+/-
Stat5a+/- Stat5b-/-
46%
8%
12%
38%
10%
15%
15%
16%
40%
CD62L - PE-Cy7
CD44 - APC
B
IL-7 signature CD8+ Tnv
IL-15 signature CD8+ Tnv
IL-7 signature CD8+ Tcm
IL-15 signature CD8+ Tcm
HGT qValue
CD8 Nv
CD8 Nv +7
CD8 Nv +15
CD8 Tcm
CD8 Tcm +7
CD8 Tcm +15
CD8 Tem
CD8 Tem +15
CD4 Nv
CD4 Nv +7
CD4 Tcm
CD4 Tcm +7
AAB vs WT
BBA vs WT

Figure S4

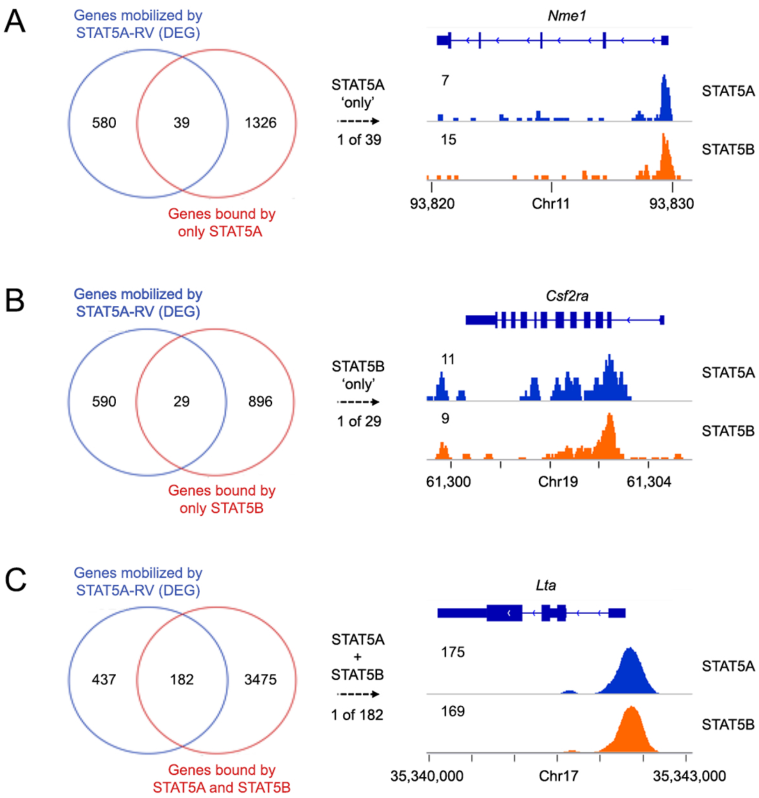

A
Genes mobilized by STAT5A-RV (DEG)
580
39
1326
Genes bound by only STAT5A
STAT5A 'only'
1 of 39
Nme1
7
STAT5A
15
STAT5B
93,820
Chr11
93,830
B
Genes mobilized by STAT5A-RV (DEG)
590
29
896
Genes bound by only STAT5B
STAT5B 'only'
1 of 29
Csf2ra
11
STAT5A
9
STAT5B
61,300
Chr19
61,304
C
Genes mobilized by STAT5A-RV (DEG)
437
182
3475
Genes bound by STAT5A and STAT5B
STAT5A + STAT5B
1 of 182
Lta
175
STAT5A
169
STAT5B
35,340,000
Chr17
35,343,000

Figure S5

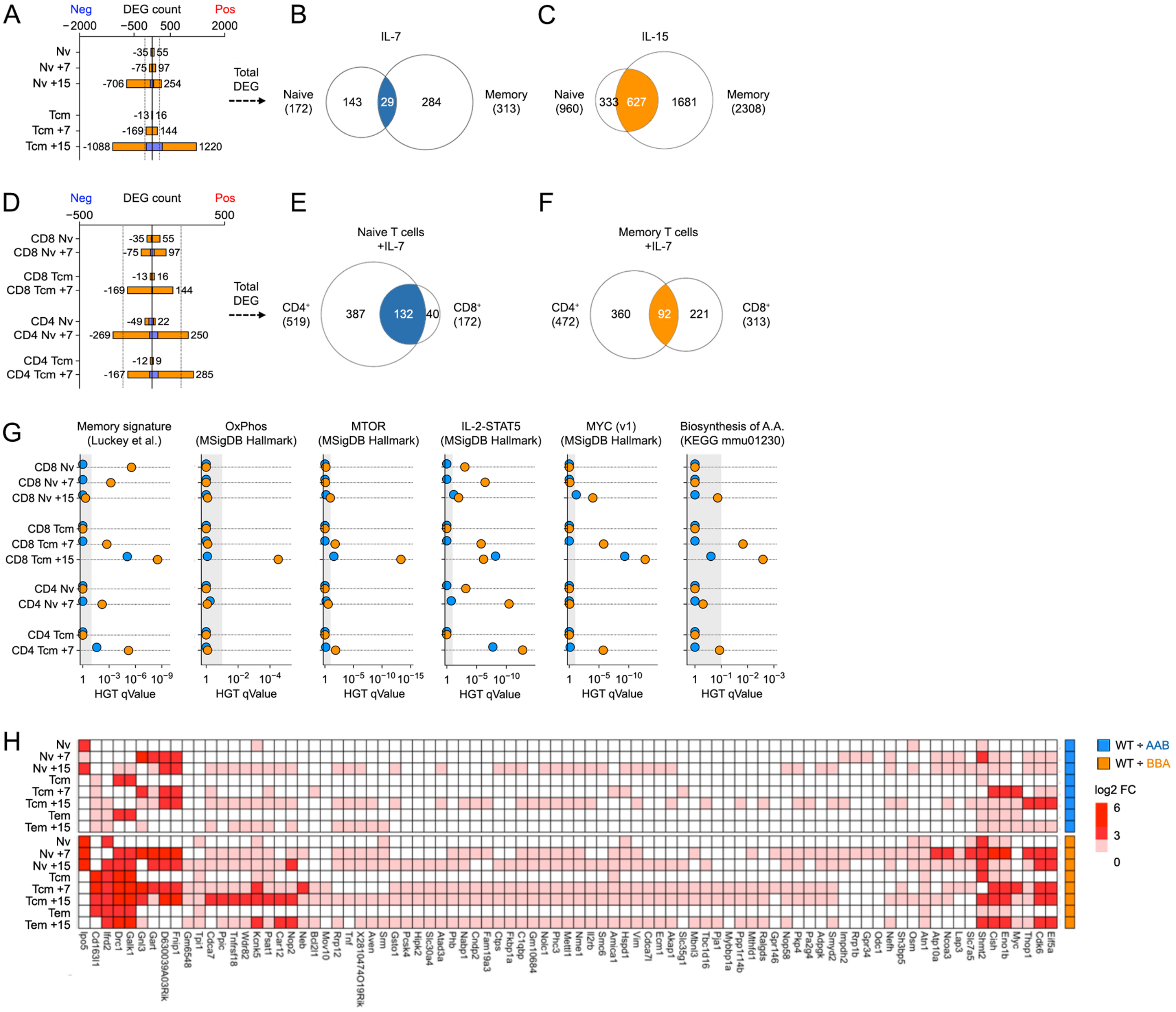

A
Neg
DEG count
Pos
Total DEG
B
IL-7
Naive (172)
Memory (313)
C
IL-15
Naive (960)
Memory (2308)
D
E
Naive T cells +IL-7
F
Memory T cells +IL-7
G
Memory signature (Luckey et al.)
OxPhos (MSigDB Hallmark)
MTOR (MSigDB Hallmark)
IL-2-STAT5 (MSigDB Hallmark)
MYC (v1) (MSigDB Hallmark)
Biosynthesis of A.A. (KEGG mmu01230)
HGT qValue
H
WT + AAB
WT + BBA
log2 FC

Figure S6

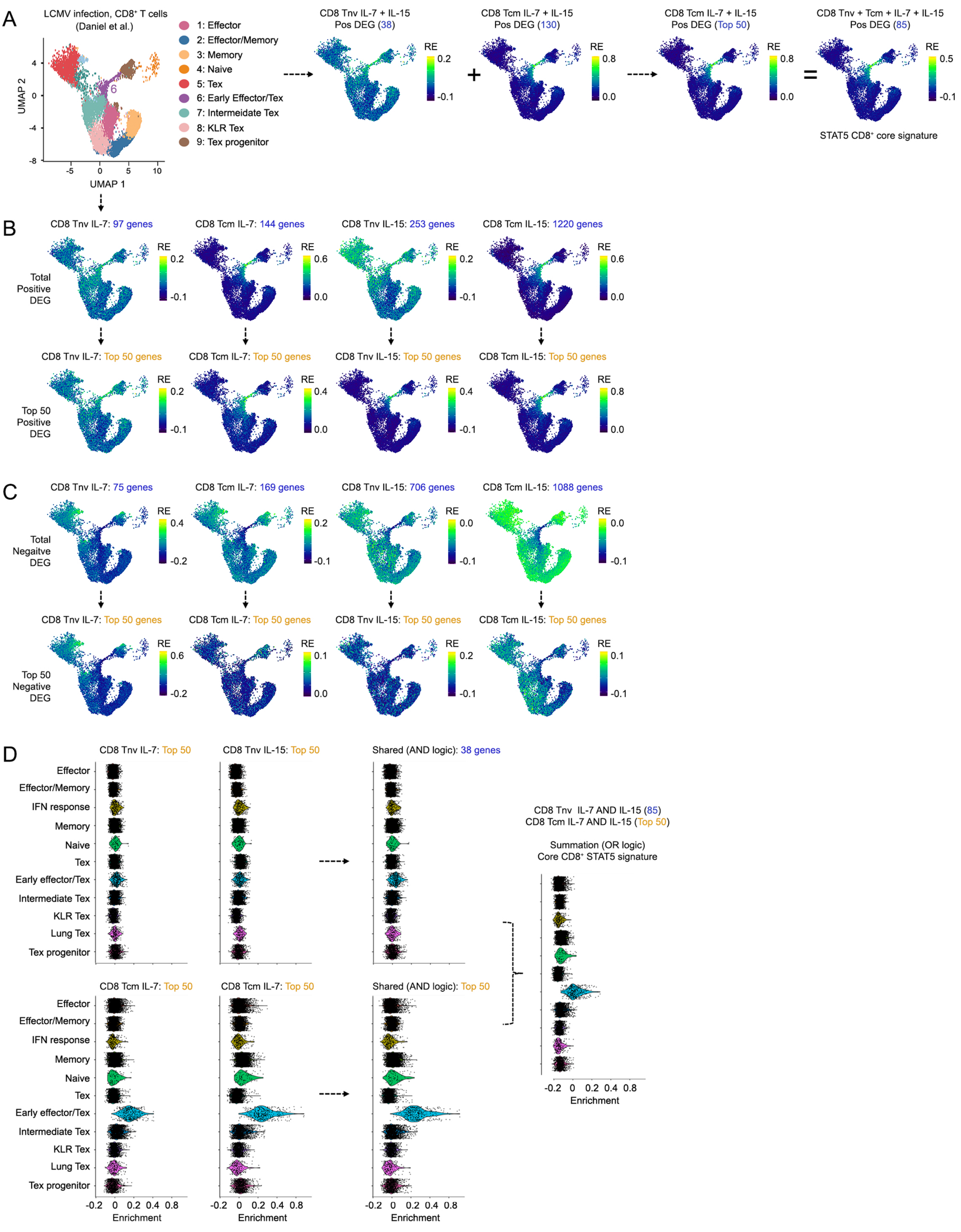
A
LCMV infection, CD8⁺ T cells
(Daniel et al.)
1: Effector
2: Effector/Memory
3: Memory
4: Naive
5: Tex
6: Early Effector/Tex
7: Intermeidate Tex
8: KLR Tex
9: Tex progenitor
UMAP 2
UMAP 1
CD8 Tnv IL-7 + IL-15
Pos DEG (38)
CD8 Tcm IL-7 + IL-15
Pos DEG (130)
CD8 Tcm IL-7 + IL-15
Pos DEG (Top 50)
CD8 Tnv + Tcm + IL-7 + IL-15
Pos DEG (85)
RE
STAT5 CD8⁺ core signature
B
CD8 Tnv IL-7: 97 genes
CD8 Tcm IL-7: 144 genes
CD8 Tnv IL-15: 253 genes
CD8 Tcm IL-15: 1220 genes
Total
Positive
DEG
CD8 Tnv IL-7: Top 50 genes
CD8 Tcm IL-7: Top 50 genes
CD8 Tnv IL-15: Top 50 genes
CD8 Tcm IL-15: Top 50 genes
Top 50
Positive
DEG
C
CD8 Tnv IL-7: 75 genes
CD8 Tcm IL-7: 169 genes
CD8 Tnv IL-15: 706 genes
CD8 Tcm IL-15: 1088 genes
Total
Negaitve
DEG
CD8 Tnv IL-7: Top 50 genes
CD8 Tcm IL-7: Top 50 genes
CD8 Tnv IL-15: Top 50 genes
CD8 Tcm IL-15: Top 50 genes
Top 50
Negative
DEG
D
CD8 Tnv IL-7: Top 50
CD8 Tnv IL-15: Top 50
Shared (AND logic): 38 genes
Effector
Effector/Memory
IFN response
Memory
Naive
Tex
Early effector/Tex
Intermediate Tex
KLR Tex
Lung Tex
Tex progenitor
CD8 Tcm IL-7: Top 50
CD8 Tcm IL-7: Top 50
Shared (AND logic): Top 50
CD8 Tnv IL-7 AND IL-15 (85)
CD8 Tcm IL-7 AND IL-15 (Top 50)
Summation (OR logic)
Core CD8⁺ STAT5 signature
Enrichment

Figure S7
