# Supplementary material for: Asymmetry and redundancy of STAT5 paralogs across CD8+ T cell differentiation states
Source: Commun Biol. 2026 Apr 4;9:529. doi: 10.1038/s42003-026-09999-9 (PMC13083952; doi:10.1038/s42003-026-09999-9)
Supplement: Supplementary file 2 — Description of Additional Supplementary Files [file 42003_2026_9999_MOESM2_ESM.docx]

**Description of Additional Supplementary Files**

**File name:** Supplementary Data 1

**Description:** Gene set catalogue.

**File name:** Supplementary Data 2

**Description:** Gene set usage.

**File name:** Supplementary Data 3

**Description:** Low amplitude DEG.

**File name:** Supplementary Data 4

**Description:** ChIP-seq peak catalogue.

**File name:** Supplementary Data 5

**Description:** Ex vivo culture conditions.

**File name:** Supplementary Data 6

**Description:** Statistics catalogue

**File name:** Supplementary Data 7

**Description:** Plot tables.
