# Supplementary material for: Asymmetry and redundancy of STAT5 paralogs across CD8+ T cell differentiation states
Source: Commun Biol. 2026 Apr 4;9:529. doi: 10.1038/s42003-026-09999-9 (PMC13083952; doi:10.1038/s42003-026-09999-9)
Supplement: Supplementary file 4 — Reporting Summary [file 42003_2026_9999_MOESM4_ESM.pdf]

nature portfolio

Corresponding author(s): Alejandro Villarino

Last updated by author(s): Aug 21, 2025

Reporting Summary

Nature Portfolio wishes to improve the reproducibility of the work that we publish. This form provides structure for consistency and transparency in reporting. For further information on Nature Portfolio policies, see our Editorial Policies and the Editorial Policy Checklist.

Statistics

For all statistical analyses, confirm that the following items are present in the figure legend, table legend, main text, or Methods section.

n/a	Confirmed	
☐	☒	The exact sample size (*n*) for each experimental group/condition, given as a discrete number and unit of measurement
☒	☐	A statement on whether measurements were taken from distinct samples or whether the same sample was measured repeatedly
☐	☒	The statistical test(s) used AND whether they are one- or two-sided<br>*Only common tests should be described solely by name; describe more complex techniques in the Methods section.*
☒	☐	A description of all covariates tested
☐	☒	A description of any assumptions or corrections, such as tests of normality and adjustment for multiple comparisons
☐	☒	A full description of the statistical parameters including central tendency (e.g. means) or other basic estimates (e.g. regression coefficient) AND variation (e.g. standard deviation) or associated estimates of uncertainty (e.g. confidence intervals)
☐	☒	For null hypothesis testing, the test statistic (e.g. *F*, *t*, *r*) with confidence intervals, effect sizes, degrees of freedom and *P* value noted<br>*Give P values as exact values whenever suitable.*
☒	☐	For Bayesian analysis, information on the choice of priors and Markov chain Monte Carlo settings
☒	☐	For hierarchical and complex designs, identification of the appropriate level for tests and full reporting of outcomes
☐	☒	Estimates of effect sizes (e.g. Cohen's *d*, Pearson's *r*), indicating how they were calculated

Our web collection on statistics for biologists contains articles on many of the points above.

Software and code

Policy information about availability of computer code

Data collection

Software used for data collection are listed under relevant sub-headings in the Methods section of the manuscript.

Data analysis

Only freely available linux- and R-based analysis packages were used. Each is listed below and under relevant sub-headings in the Methods section of the manuscript.

tophat2 2.1.1
bowtie2 2.4.0
htseq-count 2.0.3
HTSFilter 1.32.0
edgeR 3.34.0
clusterprofiler 4.0.0
pheatmap 1.0.13
macs2 2.2.9.1
homer 4.11
igv 2.15.13
ggplot2 3.3.5
datagraph 5.0
eulerr 7.0.2

For manuscripts utilizing custom algorithms or software that are central to the research but not yet described in published literature, software must be made available to editors and reviewers. We strongly encourage code deposition in a community repository (e.g. GitHub). See the Nature Portfolio guidelines for submitting code & software for further information.

Data

Policy information about availability of data

All manuscripts must include a data availability statement. This statement should provide the following information, where applicable:

- Accession codes, unique identifiers, or web links for publicly available datasets
- A description of any restrictions on data availability
- For clinical datasets or third party data, please ensure that the statement adheres to our policy

All unprocessed data will be made available via public repositories and databases. All processed data will be made available by the authors upon request.

Research involving human participants, their data, or biological material

Policy information about studies with human participants or human data. See also policy information about sex, gender (identity/presentation), and sexual orientation and race, ethnicity and racism.

Reporting on sex and gender	N/A
Reporting on race, ethnicity, or other socially relevant groupings	N/A
Population characteristics	N/A
Recruitment	N/A
Ethics oversight	N/A

Note that full information on the approval of the study protocol must also be provided in the manuscript.

Field-specific reporting

Please select the one below that is the best fit for your research. If you are not sure, read the appropriate sections before making your selection.

[x] Life sciences   [ ] Behavioural & social sciences   [ ] Ecological, evolutionary & environmental sciences

For a reference copy of the document with all sections, see nature.com/documents/nr-reporting-summary-flat.pdf

Life sciences study design

All studies must disclose on these points even when the disclosure is negative.

Sample size	Ex vivo studies included at least 4 mice per group (i.e. biological replicates) assayed over at least 3 trials (i.e. experimental repeats). In vitro studies included at least 2 mice per group assayed over at least 2 trials. These minimums were chosen to accommodate statistical analysis by t test or ANOVA. RNA-seq studies included either 2 or 3 mice per group assayed over 2 or 3 trials. Both scenarios accommodate robust quasi-likelihood F testing, as described in the Methods section of the manuscript. Total number of replicates and repeats and for each set of experiments is listed in Supplemental Table 6.
Data exclusions	No data were excluded.
Replication	To ensure reproducibility, all transgenic animal strains were inbred to a uniform genetic background (C57Bl/6), then co-housed under specific pathogen free conditions. Whenever possible littermates were used for experiments. All reagents were validated by either manufacturers or in house.
Randomization	Animals were selected for experimental groups based on genotypes, with different sets of experiments requiring different groups of genotypes.
Blinding	Experimental groups were not blinded because genotypes had to be pre-determine in order to know which (and how many) of each would be available for a given experiment.

Reporting for specific materials, systems and methods

We require information from authors about some types of materials, experimental systems and methods used in many studies. Here, indicate whether each material, system or method listed is relevant to your study. If you are not sure if a list item applies to your research, read the appropriate section before selecting a response.

Materials & experimental systems

n/a	Involved in the study
☐	☒ Antibodies
☒	☐ Eukaryotic cell lines
☒	☐ Palaeontology and archaeology
☐	☒ Animals and other organisms
☒	☐ Clinical data
☒	☐ Dual use research of concern
☒	☐ Plants

Methods

n/a	Involved in the study
☒	☐ ChIP-seq
☐	☒ Flow cytometry
☒	☐ MRI-based neuroimaging

Antibodies

Antibodies used

Target, fluorochrome and clone for all antibodies used are listed below. This information will be added to the Methods section upon revision, along with Fluorochrome labels for relevant figures.

CD4 - Pacific Orange - ThemoFisher, clone: RM4-5, cat#: MCD0430
CD8a - APC-Cy7 - Biolegend, clone: 53-6.7, cat#: 100714
CD3e - PerCP-Cy5.5 - Biolegend, clone: 145-2C11, cat#: 100328
CD45.1 - Pacific Blue - Biolegend, clone: A20, cat#:110722
CD45.2 - PE/Dazzle 594 - Biolegend, clone: 104, cat# 109845
CD44 - APC - Biolegend, clone: IM7, cat#: 103012
CD62L - PE-Cy7 - Thermo, clone: MEL-14, cat#: 25-0621-82
CD127 (IL-7R) - PE-Cy7 - Biolegend, clone: A7R34, cat#: 135014
GZM-A - APC - ThemoFisher, clone: GzA-3G8.5, cat#: 17-5831-82
IL-2Ra - PE - Biolegend, clone: PC61, cat#: 102007
Eomes - e450 - Thermo, clone: Dan11mag, cat#: 48-4875-82
Tbet - PE-Cy7 - invitrogen, clone: 4B10, cat#: 25-5825-80
CD19 - Af700 - BD, clone: 1D3, cat#: 557958

Validation

Specificity of all antibodies was validated by manufacturers and further verified through positive and negative controls built into all assays.

Animals and other research organisms

Policy information about studies involving animals; ARRIVE guidelines recommended for reporting animal research, and Sex and Gender in Research

Laboratory animals

Species, strain, and age of all experimental animals is detailed in the Methods section of the manuscript.

Wild animals

N/A

Reporting on sex

As noted in the Methods section, both female and male mice were used. We found no evidence that sex influence the reported findings.

Field-collected samples

N/A

Ethics oversight

All necessary approvals and oversight are described in the Methods section of the manuscript

Note that full information on the approval of the study protocol must also be provided in the manuscript.

Plants

Seed stocks

N/A

Novel plant genotypes

N/A

Authentication

N/A

Flow Cytometry

Plots

Confirm that:

☒ The axis labels state the marker and fluorochrome used (e.g. CD4-FITC).

☒ The axis scales are clearly visible. Include numbers along axes only for bottom left plot of group (a 'group' is an analysis of identical markers).

☒ All plots are contour plots with outliers or pseudocolor plots.

☒ A numerical value for number of cells or percentage (with statistics) is provided.

Methodology

Sample preparation	Samples for surface, intracellular and intranuclear cytometry were prepared using standard protocols, as described in the Methods section.
Instrument	All cytometry data was collected on 4- or 5-laser Cytek Aurora cytometers.
Software	All cytometry data was analyzed using FlowJo 10.10.
Cell population abundance	CD4+ and CD8+ cells are abundant in lymphoid tissues and easily identified by surface expression of these markers. For retroviral transduction experiments, only GFP+ cells were considered, typically constituting 20-50% of all cells.
Gating strategy	All gating strategies are described in the Results, Methods and/or Figure Legends.

☒ Tick this box to confirm that a figure exemplifying the gating strategy is provided in the Supplementary Information.
